# Adherence to hepatitis B vaccination recommendations for children and adolescents aged 3 to 17 years in Germany, 2014–2017: results from a cross-sectional national population-based study

**DOI:** 10.1186/s12879-026-12519-z

**Published:** 2026-01-16

**Authors:** Sofie Gillesberg Lassen, Thomas Harder, Sandra Dudareva, Klaus Stark, Christina Poethko-Müller

**Affiliations:** 1 Department of Infectious Disease Epidemiology, Robert Koch Institute, Seestraße 10, 13353 Berlin, Germany; 2 Charité Universitätsmedizin Berlin, Berlin, Germany; 3 Institute of Public Health, Riga Stradins University, Riga, Latvia; 4 Department of Epidemiology and Health Monitoring, Robert Koch Institute, Berlin, Germany

**Keywords:** Hepatitis B, Vaccination, Timelines, Cross-sectional study, Population-based

## Abstract

**Background:**

Although universal childhood vaccination of hepatitis B has been recommended in Germany since 1995, evidence on adherence to the recommended hepatitis B vaccination schedule is lacking. Using data from the second wave of the population-based German Health Interview and Examination Survey for Children and Adolescents (KiGGS) (2014–2017), we aimed to assess the adherence to the recommended hepatitis B vaccination schedule by describing the timeliness of the first dose, the timeliness of the recommended vaccination series, the vaccination series by number of doses, and the adherence to six months period between the last two doses given.

**Methods:**

Reflecting recommendations in Germany, we defined a timely first dose as given at two months of age or as a birth-dose given prior to two days of age. A catch-up vaccination series was defined as first dose given after 14 months of age. We defined a recommended hepatitis B vaccination series as at least three doses, with six months between the two last doses, as in accordance with national recommendations. We defined a hepatitis B vaccination series as being timely if the last dose was given prior to 15 months of age. We calculated weighted proportions and their 95% confidence intervals (CI) overall, according to socio-demographic and health utilization characteristics.

**Results:**

Of the 3,165 included participants, 93.4% [95%CI: 91.9%-94.5%] received at least one hepatitis B vaccination dose; 48% [95%CI: 45%-51%] received a timely first dose and 4.62% [95%CI 3.59%–5.92%] received a catch-up vaccination series. Of all participants, 79.1% [95%CI: 76.8%-81.2%] received a recommended series, and 30.9% [95%CI: 28.3%-33.6%] had a timely recommended series. We found a higher proportion of children in birth-cohorts after 2001 with a timely first dose and a recommended series than children born in 1994–2001. The proportion of children with a timely first dose was higher for children living in eastern Germany (66.3%; 95%CI: 61.1%–71.2%) compared with western Germany (46.2%; 95%CI: 42.9%–49.6%).

**Conclusions:**

Our analyses reveal gaps in adherence to vaccination recommendations against hepatitis B in Germany. However, we identified great potential for catch-up vaccinations with close to 95% of children and adolescents receiving at least one hepatitis B vaccination dose.

**Supplementary Information:**

The online version contains supplementary material available at 10.1186/s12879-026-12519-z.

## Background

Viral hepatitis including infections with hepatitis B virus (HBV) is a major public health threat globally while the epidemiological situation differs between countries [1]. In Europe, infections with HBV is one of leading causes of chronic liver disease, liver cirrhosis, and hepatocellular carcinoma, resulting in large burdens of disease [2, 3]. However, highly effective measures are available to prevent HBV, and WHO has a goal of eliminating viral hepatitis as a public health treat by 2030 [1, 4]. For HBV infections, prevention strategies include childhood vaccination and preventing mother-to-child transmission. Targets for HBV elimination in the WHO European Region include (1) 95% coverage with three doses of HBV vaccination in countries that implement universal childhood vaccination and (2) 90% coverage with interventions to prevent mother-to-child transmission of HBV including, screening of pregnant women and post-exposure-prophylaxis of children born to HBV positive women, or universal HBV vaccination at birth [5].

In Germany, HBV vaccination based on risk indication was introduced in 1982 [6]. Since 1995, the German Standing Committee on Vaccination (STIKO) recommends universal childhood HBV vaccination [7]. Hexa-valent vaccines including a hepatitis B component were introduced in 2000 [8]. In March 2001, the recommended vaccination series for children born to Hepatitis B Surface antigen (HBsAg) negative women was changed to either (a) three monovalent or non-pertussis containing doses at the ages of two, four, and 11–14 months or (b) four doses of poly-valent pertussis containing vaccine at two, three, four, and 11–14 months of age [9]. For both series the two last doses should have at least six months between them [9]. In August 2020, the recommendation for a regular vaccination series was changed to three doses at two, four, and 11 months of age, regardless of the vaccine containing pertussis or not, and the series should be finalised prior to one year of age [10–12]. At the introduction of HBV-vaccinations catch-up vaccination was recommended until 15 years of age until 1998 where it was changed to 18 years of age [7, 11, 13]. Coverage of immunisation costs by health insurance is stipulated by the Federal Joint Committee based on the STIKO recommendations [14]. Checking the vaccination status and guidance on improving the vaccination protection of a child is part of the well-care health services in Germany [15–17].

Screening of pregnant women for HBV-infections and post-exposure prophylaxis of infants born to HBsAg-positive women was introduced in 1994 [18]. According to the recommendation for post-exposure prophylaxis, HBV immunoglobulin should be given within 12 hours after birth, together with a monovalent HBV vaccination (birth-dose). The vaccination series should be completed by two consecutive doses four weeks and five months later or with a dose at four weeks, two and 12 months. Furthermore, a birth dose is recommended for infants born to women with unknown HBsAg status [19].

In 2013–2018 the coverage of HBV vaccinations (complete series) based on school health examinations was found to range between 86.9% and 88.3%. For children born in 2016 a vaccination coverage of 75.5% [95%CI: 68.1%-81.0%] at 24 months of age was estimated using statutory health insurance data [20]. An analysis done on statutory health insurance data prior to changing the vaccination recommendations in 2020 showed that 42% of children born in 2014 with information on all vaccination dates and who could be observed up 24 months of age had finished an either three or four dose series at 15 months of age [10]. Since the introduction of HBV vaccination there has been a gap between the DPT and HBV vaccination coverage. Only since 2015, the gap is closing and for children born in 2021 the difference in coverage was 2%-points at 24 months of age (77% for DPT and 75% for hepatitis B) [21].

Neither way of reporting the vaccination coverage includes descriptions on the adherence to recommendations in terms of timeliness of the first and last dose of HBV vaccine given at the recommended ages. The timing between the two last doses is important for the persistence of anti-hepatitis B antibodies ≥ 10 mIU/ml [22, 23]. The current vaccination coverage reporting only partly report on the adherence regarding the required six-months between the two last doses, as only children with the correct spacing between the two last doses were considered fully vaccinated. Information on proportion of children who got the right number of doses but without the correct spacing of the two last doses is thus missing. Nor do they include catch-up vaccinations or the potential of finalising immunisation series in children who did not yet finish the vaccination series.

We aimed to assess the adherence to HBV vaccination recommendations by (1) estimating the proportion of children and adolescents aged 3–17 years with a first dose of HBV vaccination given at the recommended age in in Germany, 2014–2017 (2) estimating the proportion of children and adolescents with a documented HBV vaccination series according to the number and type (pertussis containing or not) of doses, the time between the last two doses, and if the last dose is given at the recommended age. Finally, we aimed to describe adherence to recommendations by socio-demographic characteristics and utilisation of preventive health services to identify gaps and opportunities to improve HBV vaccination coverage as well as the timeliness of the first dose.

## Methods

### Data source

The second wave of the “German Health Interview and Examination Survey for Children and Adolescents” (KiGGS) was conducted by Robert Koch Institute in 2014–2017, using a national population-based sample of children and adolescents aged 0 to 17 years residing in Germany. In a randomly allocated sample of the cross-sectional study, information on children aged 3 to17 years including details on vaccinations was collected [24, 25].

We initially included 3,567 participants of the KiGGS Wave 2 for whom details on vaccination was collected. We excluded participants with at least one unknown vaccination date, unreadable or incomplete vaccination cards (supplementary Fig. 1).

Information on socio-demographic characteristics and utilization of children and adolescent well-care health services were self-reported using structured questionnaires. Due to data protection, date of birth was reduced to month and year in the dataset. Information on type and date of vaccination was collected from vaccination cards and entered in a data base after completion of data collection using double entry strategies and plausibility checks to ensure high data quality. We performed additional vaccination data quality checks specific for HBV vaccinations looking at the original data for participants with implausible vaccination dates or with a first dose given prior to two months of age.

### Definition of timeliness

In order to assess the timeliness of the first dose we defined a birth-dose, a timely first dose, and categorized late first doses according to whether they lead to delayed, catch-up, or postponed vaccination series.

- As an approximation for a birth-dose given prior to two days of age, we defined a timely birth-dose as a first dose with monovalent HBV vaccine given within the 1st of the birth month and year until 1st of the following month (supplementary Fig. 2a).
- We defined a timely first dose as a birth-dose or a first dose given at two months of age, calculated as a first dose given on the first day in the second month after the month of birth until last day of the month following (supplementary Fig. 2b).
- A first dose leading to a delayed vaccination series was defined as a first dose too late for the last dose of a series to be given prior to turning 15 months of age (supplementary Fig. 2b).
- A first dose compatible with catch-up vaccination series was defined as a first dose given after 14 months of age and calculated as a first dose given after the last possible day of being 14 months of age (supplementary Fig. 2b).
- A first dose was set to lead to a postponed vaccination series when given at five years of age or older. The proportion of first dose in a postponed series was calculated as a first dose given after the last possible day of being four years of age in a subsample of 2,765 participants aged five years or older (supplementary Fig. 2b).

### Vaccination series classification

We defined a recommended vaccination series as three or more doses of either mono- or polyvalent HBV containing vaccines with at least six months between the two last doses, as recommended. A recommended vaccination series was defined as timely when the last dose was given prior to 15 months of age. All timeliness definitions were done to reflect STIKO recommendations at the time of the study.

### Statistical analysis

We calculated weighted proportions of all included participants and their 95% confidence intervals (CI) to correct for regional differences in the distribution of age structure and sex distribution within federal states and with regard to parental levels of education and German nationality status [24, 25]. Participants who received a birth-dose were described separately and excluded from other proportion estimations. The proportion of postponed vaccination series was calculated for participants aged 5 years or older only.

We described the timely first doses, recommended and timely recommended vaccination series further by sex (male, female), birth-cohort (1994–1997, 1998–2001,2002–2005, 2006–2009, 2010–2014), living in eastern Germany (federal States of former East Germany, including Berlin) or western Germany (federal states of the former West Germany), municipality size (rural: <5000 inhabitants, small town: 5000 < 20,000 inhabitants, medium-sized town: 20,000-<100,000 inhabitants, urban: >=100,000), CASMIN educational level of the mother (low, middle, high), being the firstborn (yes, no), migration background (none, one-sided, two-sided), and attending all early childhood development health service well-care visits until age 24-months (yes, no). For the description by birth cohort we combined the birth cohorts 2010–2013 and 2014–2017, as fewer than five participants were included from birth cohort 2014–2017, due to inclusion criteria of three years of age at time of data collection. Participants with a missing value were excluded from the weighted proportion calculations for the socio-demographic and health care utilization characteristics. We also described the recommended series by vaccine type (pertussis or non-pertussis containing vaccine), number (three, four, more than four) of vaccination doses, and calculated the mean number of days between doses and their interquartile range (IQR).

Differences between groups were described based on the weighted proportions and their 95% CI. Proportions were considered statistically different if their 95%CI did not overlap. When comparing included participant with the original sample with information on vaccination we calculated the mean age of participants and its IQR. We described the characteristics of the included participants and the original sample by calculating crude proportions. We performed all analyses using Stata 17.0 (StataCorp, USA) using svyset for the weighted analysis.

## Results

**Fig. 1:**
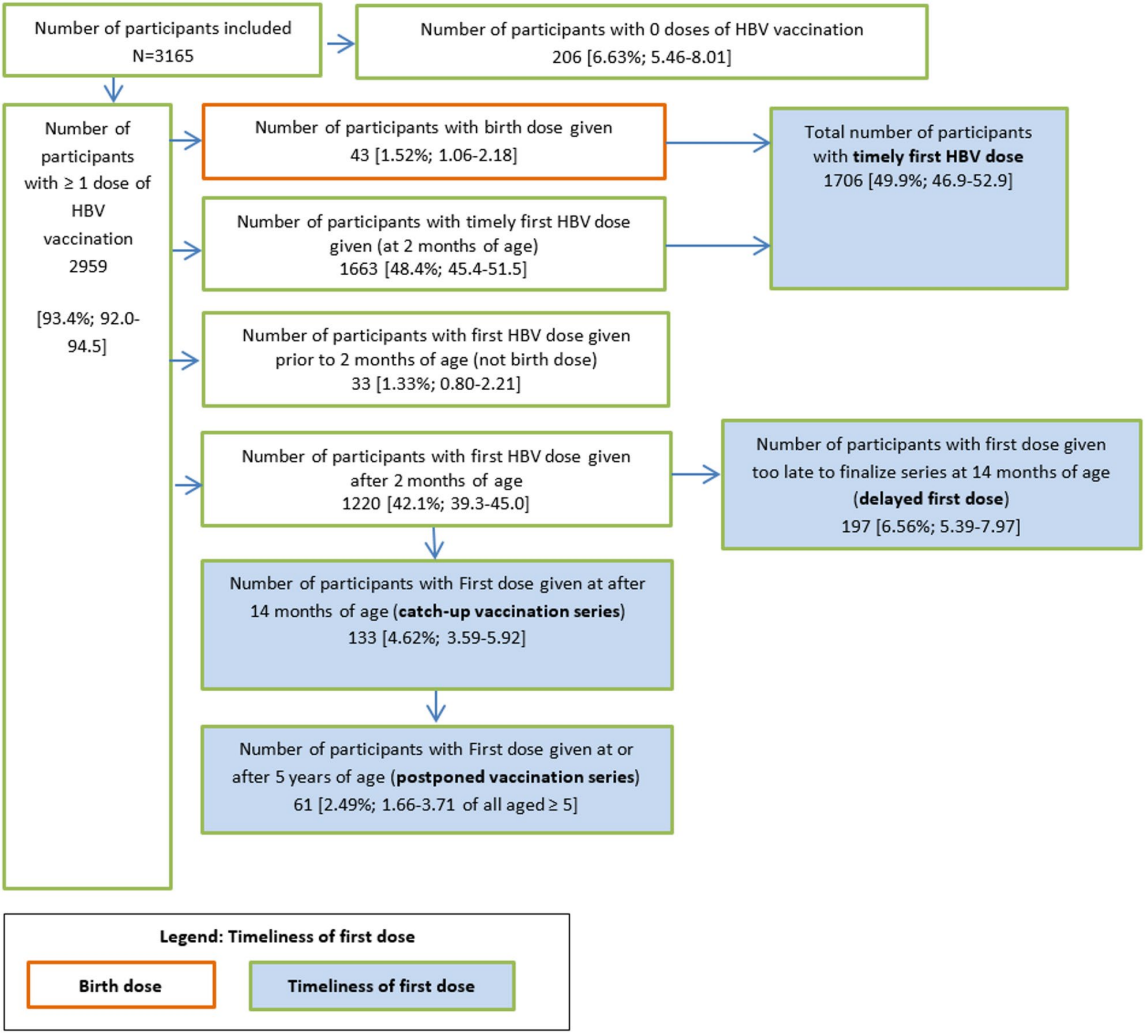
Flow-chart of participants by the timeliness of their first HBV vaccination dose; crude number of participants and the weighted proportion including its 95%CI of children and adolescents aged 3–17 years, 2014–2017, Germany, *n* = 3165

We included 3,165 participants in the analysis. Of the 402 excluded participants, 329 were excluded due to not presenting the vaccination card or their vaccination cards being partly unreadable or incomplete, and 73 had one or more dates of HBV vaccination missing (supplementary Fig. 1). Of the included participants 2,959 [93.4%, 95%CI: 92.0%-94.5%] had received at least one HBV vaccination (Fig. 1).

The included participants had a similar distribution to the original sample in terms of socio-demographical characteristics except for slight differences in migration background where 76.1% of included participants had no migration background compared to 74.0% of all participants. Additionally, 87.1% of the included participants had gone to all well-care visits compared to 85.0% of the original sample (Table 1).

**Table 1:** Non-weighted number and proportion of the total study population of the examination part of KiGGS Wave 2 and included participants by social and health care utilisation characteristics, *N* = 3567

	Total participants examination		Included participants	
	*n*	%	*n*	%
**Total**	3567	100	3165	100
**Sex**				
Male	1766	49.5	1567	49.5
Female	1801	50.5	1598	50.5
**Birth cohort**				
1994–1997	66	1.85	56	1.77
1998–2001	919	25.8	793	25.1
2002–2005	1016	28.9	896	28.3
2006–2009	934	26.2	842	26.6
2010–2013	630	17.7	576	18.2
2014–2017	2	0.06	2	0.06
**Geographical place of living**				
Western Germany	2338	65.6	2053	64.9
Eastern Germany (incl Berlin)	1229	34.5	1112	35.1
**Municipality Size**				
Rural (< 5000 inhabitants)	707	19.8	631	19.9
Small town (5000-<20000)	1042	29.2	930	29.4
Middle sized town (20000-<100000)	1011	28.3	904	28.6
Urban ( > = 100000)	807	22.6	700	22.1
**Educational level**,** Mother (CASMIN)**				
Low	570	16.0	507	16.0
Middle	1994	55.9	1800	56.9
High	828	23.2	745	23.5
Unknown	175	4.91	113	3.57
**First child in the family**				
No	2040	57.2	1782	56.3
Yes	1527	43.7	1383	43.7
**Migration background**				
None	2640	74.0	2409	76.1
One-sided	333	9.34	291	9.19
Two-sided	496	13.9	409	12.9
Missing	98	2.75	56	1.77
**All recommended early childhood development well-care visits until age 24-months**				
No	536	15.0	410	13.0
Yes	3031	85.0	2755	87.1

### Timely first dose

In total, 49.9% [95%CI: 46.9%-52.9%] of included participants had received a timely first dose; 1.52% [95%CI: 1.06%-2.18%] received a birth-dose and 48.4% [95%CI: 45.4%-51.5%] received a timely first dose. We found that 1.33% [95%CI: 0.80%-2.21%] of included participants had received the first dose prior to two months of age but too late to be a birth-dose with a median of 10 days (range 1–38 days) between the day the first dose was given and being two months of age. In total, 42.1% [95%CI: 39.3%-45.0%] received the first dose of HBV vaccination after the age of two months. Further differentiation showed that 6.56% [95%CI: 5.39%-7.97%] of the included participants had received a delayed vaccination series and 4.62% [95%CI: 3.59%-5.92%] had received a catch-up vaccination series. Of participants aged 5 years or older, 2.49% [95%CI: 1.66%-3.71%] had a postponed vaccination series (Fig. 1). The median number of days a first dose was given after the age of two months was 26 days, IQR:11–102 days.

### Timely first dose by demographic characteristics and preventive health care utilisation

The proportion of children, who had received a timely first dose, ranged from 22.0% [95%CI: 11.3%-38.5%] in birth-cohort 1994–1997 to 60.2% [95%CI: 53.6%-64.5%] for birth-cohort 2006–2009 and 59.2% [95%CI: 53.4%-64.8%] for birth-cohort 2010–2014. The largest difference between two consecutive birth-cohorts was 31.7% [95%CI: 27.1%-36.6%] for 1998–2001 to 53.6% [95%CI: 48.9%-58.3%] for 2002–2005 (Table 2).

**Table 2:** Weighted proportion of children and adolescents aged 3–17 years with a timely first dose, a recommended series, and a timely recommended series by socio-demographic and health care utilisation characteristics, Germany, 2014–2017

	Timely first dose *n* = 1706		Recommended series *n* = 2531		Timely recommended series *n* = 1072	
	%	95%CI	%	95%CI	%	95%CI
**Total**	49.9	46.9–52.9	79.1	76.8–81.2	30.9	28.3–33.6
**Sex**						
Male	51.0	47.1–54.8	79.7	76.5–82.6	32.3	28.9–35.8
Female	48.8	45.4–52.3	78.5	75.7–81.0	29.4	26.2–32.9
**Birth cohort**						
1994–1997	22.0	11.3–38.5	68.2	50.1–82.1	30.9	16.9–49.8
1998–2001	31.7	27.1–36.6	74.1	69.4–78.2	23.9	19.9–28.4
2002–2005	53.6	48.9–58.3	81.1	77.4–84.4	31.5	27.1–36.3
2006–2009	60.2	55.6–64.5	81.6	78.2–84.6	34.1	29.7–38.8
2010–2014*	59.2	53.4–64.8	81.1	76.7–84.3	35.3	30.7–40.2
**Geographical place of living**						
Western Germany	46.2	42.9–49.6	78.0	75.3–80.5	28.8	25.9–31.9
Eastern Germany (incl Berlin)	66.3	61.1–71.2	84.0	80.7–86.8	40.1	35.4–45.0
**Municipality Size**						
Rural (< 5000 inhabitants)	49.7	41.6–57.7	78.9	73.2–83.6	28.8	22.0-36.8
Small town (5000-<20000)	48.5	42.7–54.3	80.1	76.2–83.6	32.7	27.2–38.7
Middle sized town (20000-<100000)	50.0	44.8–55.3	78.5	73.7–82.7	31	26.7–35.7
Urban ( > = 100000)	51.4	45.7–57.1	78.8	74.4–82.6	30.1	26.0-34.5
**Mothers Level of education (CASMIN)**						
Low	50.7	44.8–56.6	78.8	74.0-82.9	26.6	21.5–32.4
Middle	52.8	49.3–56.2	81.5	78.7–83.9	33.1	30.0-36.5
High	44.1	39.2–49.1	74.2	69.7–78.4	32.2	27.7–37.1
**First child in the family**						
No	47.2	43.7–50.7	79.0	76.0-81.7	25.8	22.7–29.1
Yes	53.5	49.4–57.5	79.2	76.0-82.1	37.5	33.9–41.3
**Migration background**						
None	49.0	45.4–52.7	79.8	77.2–82.2	32.3	29.3–35.5
One-sided	49.7	42.9–56.6	81.2	74.7–86.3	29.4	22.7–37.3
Two-sided	51.9	45.9–57.9	74.6	69.6–79.1	25.9	21.4–30.9
**All recommended early childhood development well-care visits until age 24-months**						
No	43.2	36.9–49.8	69.7	63.7–75.1	23.8	18.30–30.4
Yes	51.0	47.8–54.3	80.6	78.3–82.7	32.0	29.3–35.0

*the two participants in birth cohort 2014–2017 included due to low numbers

We found a lower proportion of children in western Germany with a timely first dose, 46.2% [95%CI: 42.9%-49.6%], compared to children in eastern Germany, 66.3% [95%CI: 61.1%-71.2%] (Table 2).

For children with mothers with a higher education, 44.1% [95%CI: 40.0%-49.6%], had a timely first dose. This was lower than in children of mothers with medium-level education, 52.8% [95%CI: 49.3%-56.2%] as well as for children of mothers with low-level of education, 50.7% [95%CI: 44.8–56.6%] although the 95%CI overlap (Table 2).

Although the 95%CI slightly overlap, a higher proportion of first-born children had a timely first dose, 53.5% [95%CI: 49.4%-57.5%], compared with 47.2% [95%CI: 43.7%-50.7%] of children having at least one older sibling. Similarly, a higher proportion of children who utilised all recommended early childhood development health visits had a timely first dose, 51.0% [95%CI: 47.8%-54.3%] compared with those who did not, 43.2% [95%CI: 36.9%-49.8%]. The prevalence of a timely first dose was not different with regard to sex, migration status, and municipality size.

### Adherence to number of doses and time between last two doses

**Fig. 2:**
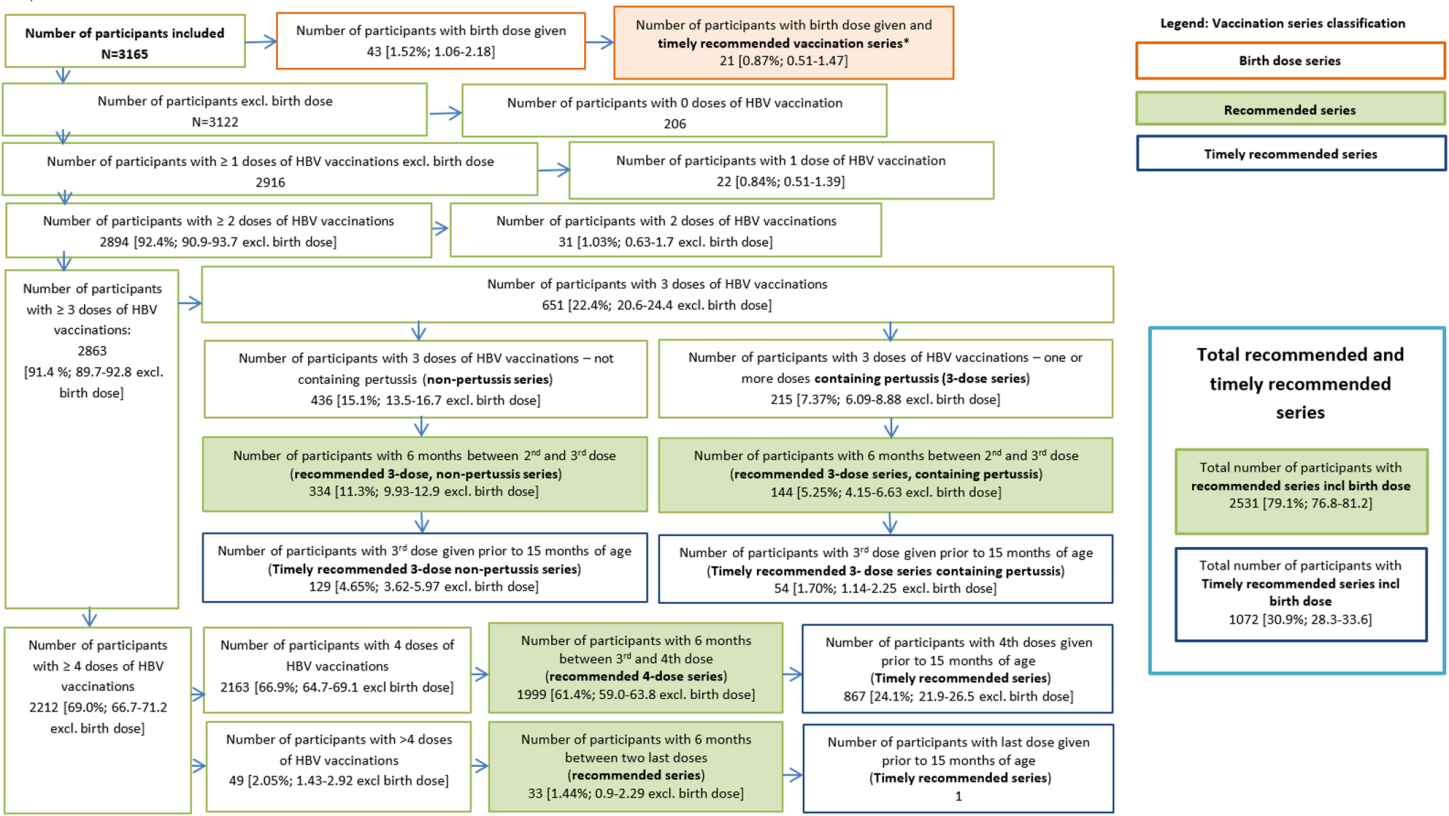
Flow-chart of the crude number of participants and weighted proportion of children and adolescents aged 3-17 years, 2014-2017 in Germany, according to number and type of doses (pertussis containing or not), and timing between the last two doses. * see supplementary figure 3

We estimated that 91.4% [95%CI: 89.7%-92.8%] of children had received at least three doses of HBV containing vaccines and 79.1% [95%CI:76.8%-81.2%] had a recommended HBV vaccination series (Fig. 2). The number and type of vaccination doses in the recommended vaccination series were distributed as follows within the 3,165 included participants: 5.25% [95%CI:4.15%-6.63%] had three doses of at least one pertussis-containing HBV vaccine, 11.3% [95%CI: 9.93%-12.9%] had three non-pertussis-containing HBV vaccines, 61.4% [95%CI 59.0%-63.8%] had received four doses of pertussis-containing HBV vaccine and 1.44% [95%CI: 0.90%-2.29%] had received more than four doses of pertussis-containing HBV vaccine (Fig. 2).

We found that 30.9% [95%CI:28.3%-33.6%] of the participants had a timely recommended vaccination series: 24.1% [95%CI:21.9%-26.5%] with four doses, 4.65% [95%CI 3.62%-5.97%] with three doses of non-pertussis containing vaccines, 1.70% [95%CI: 1.14%-2.25%] with three doses with at least one pertussis-containing vaccine and 0.87% [95%CI: 0.51%-1.47%] with a birth-dose series (Fig. 2)^1^. An estimated 0.84% [95%CI: 0.51%-1.39%] of the participants had received one HBV containing vaccine dose only, and 1.03% [95%CI: 0.63%-1.7%] had received two HBV containing vaccine doses only (Fig. 2).

### Adherence to number of doses and time between last two doses by demographic characteristics and preventive health care utilisation

We did not observe any differences regarding the proportion of participants who has received a recommended series by sex or birth cohort, although the proportion increased from 74.1% [95%CI: 69.4%-78.2%] for birth cohort 1998–2001 to 81.1% [95%CI: 77.4%-84.4%] for 2002–2005 (Table 2). Also, no difference was seen for migration background. Although the proportion of children with a two-sided migration background with a recommended series, 74.6% [95%CI: 69.6%-79.1%], was lower than for children with no (79.8%; 95%CI: 77.2%-82.2%) or one-sided migration background (81.2%; 95%CI: 74.7%-86.3%), the 95%-CI overlapped (Table 2).

We found the proportion with a recommended vaccination series was lower in western Germany, 78.0% [95%CI: 75.3%-80.5%], than in eastern Germany, 84.0% [95%CI: 80.7%-86.8%]. The difference being more pronounced for timely recommended series with 28.8% [25.9%-31.9%] in western Germany versus 40.1% [35.4%-45.0%] in eastern Germany.

Adherence to the HBV vaccination recommendation was higher in children who took part in all recommended early childhood development well-care visits until two years of age, 80.6% [95%CI: 78.3%-82.7%], than in children who had missed one or more health examination, 69.7% [95%CI: 63.72%-75.10%]. This difference was also seen for timely recommended series (Table 2).

First-born children had a higher proportion of timely recommended series, 37.5% [95%CI: 33.9%-41.3%], than children who were not first-born, 25.8% [95%CI: 22.7%-29.1%], however, the proportion of first-borns and non-first-borns was not different for a recommended series (Table 2).

A lower proportion of children of mothers with a high level of education had a recommended vaccination series, 74.2% [95%CI: 69.7%-78.4%] than children of mother’s middle level of education 81.5% [95%CI: 78.7%-83.9%], although their 95%CI were almost overlapping (Table 2). The difference between education levels was not observed when looking at timely recommended series (Table 2). The proportion of children following the recommended HBV vaccination series was not different with regard to municipality size.

## Discussion

To our knowledge this is the first study investigating the adherence to HBV vaccination recommendations in terms of timeliness of the first dose, the number of doses, and time between the last two doses in a population-based sample of children and adolescents in Germany.

We found that about half of children aged 3–17 in 2014–2017 received a timely first dose. Considering that only 5% had a delayed series and 3% had a catch-up series, the delay of the first dose likely happens due acute illness, forgetting that it is time to vaccinate, or other reasons for shorter delays in receiving a vaccination dose. However, we do not have any data to support these suggested reasons for delays in receiving the first dose in our data.

Our study is to our knowledge also the first study to estimate of the proportion of children in the birth-cohorts 1994–2014 with a recommended three dose series with polyvalent vaccines. For 20 years, between 2001 and 2021, the German recommendations included four-doses of the polyvalent (pertussis containing) HBV vaccines, which differed to the HBV vaccination recommendations from WHO including three doses regardless of type of vaccine. This led to children with three polyvalent HBV vaccine doses and six months between the last two doses to be disregarded as having a recommended vaccination series and thus categorised as unvaccinated when calculating the vaccination coverage. This would have led to a lower vaccination coverage than if calculated based on the WHO recommended three dose series, which particular for comparison with other counties would have underestimated the HBV vaccination coverage of Germany.

Our results confirm that in 2014–2017, less than 95% of children and adolescent aged 3–17 years in Germany had a recommended HBV vaccination series according to the definition in this study. However, we identified opportunities to improve completeness of vaccination series and adherence to recommendations in terms of timeliness of doses. Our results show a high willingness to be vaccinated against HBV infection with 93.4% of children and adolescents having received at least one HBV vaccination dose and more than 90% vaccinated with at least 3 doses. However, about 10% got the last dose prior to the recommended six months after the previous dose. One reason for this could be that an additional dose was originally planned according to the four-dose series recommended by STIKO until 2020 [9–11]. In addition, only 30.9% had a timely recommended series, which shows potential to firstly improve the timing between the two last doses and secondly finishing the vaccination series within the recommended age-span.

Our estimate for children with a recommended series (81.2%) is lower than the vaccination coverage found though the school health entry examinations, which ranged from 86.9% to 87.6% in the years 2015–2020 [26]. However, looking at the number of doses only, we found a higher proportion of children with three or more doses (91.4%). Differences may be explained by methodological issues as definition of a complete vaccination series vary between data sources of the 16 federal states in Germany and when using statutory health insurance data [20]. The definition can vary in terms of number of doses only or with six months between the last two doses irrespectively of three or four doses. We included 5% of participants with only three doses of polyvalent (pertussis containing) HBV vaccine, which are not included in the vaccination coverage based on the school health entry examinations for children born prior to the 2021 change in vaccination recommendation. When using the statutory health insurance and including three dose vaccination series with any hepatitis B containing vaccine and 6 months between the two last doses a vaccination coverage of 75.5% at the age of 24 months for children born in 2016 and 82.2% at age 36 months for children born in 2015 was found [20]. This corresponds well with our estimate for recommended series.

The proportion of children with a recommended series in our study is low when comparing with the coverage of DPT vaccinations in children for children with the birth cohorts 2010–2013 when using the school health examinations [20]. However, when comparing with the coverage for children born in 2016 using health insurance data, the proportion of children with a recommended HBV series corresponds well with the coverage of tetanus vaccination at 78% [20]. This is also in line with the latest vaccination coverage reports showing the gap between DPT and HBV vaccination close [21].

We found a lower proportion of children with a timely first dose, a higher proportion of postponed series, and lower proportion of recommended series in western Germany than in eastern Germany showing geographical differences in adherence to vaccination recommendations. When comparing with children born in 2021 at 24 months of age the regional difference in vaccination coverage is not as strong [27]. Whether the difference between western and eastern Germany observed in our study is due to differences in adherence by doctors or parents or remining cultural differences after re-unification is not possible to say from our data. In a stratified sub-analysis (data not shown) we did observe a higher proportion of children who had participated in all well-child visits having a timely first dose (66.3%; 95%CI: 60.7%-71.5%) or recommended vaccination series (85.4%; 95%CI: 82.2%-88.1%) in eastern Germany than western Germany (timely first dose; 47.5%, 95%CI: 43.8%-51.2%, Recommended vaccination series; 79.5%, 95%CI: 76.6%-82.0%). This indicates that some structural differences in adherence to vaccine recommendations between eastern and western federal states may still persist. A structural healthcare factor such as physician density does not seem to explain the difference as the eastern federal states have a higher number of people per doctor than the western federal states [28]. However, the data may support the findings from 2016 that parents in eastern federal states more often see a need for their child to be vaccinated against diphtheria, tetanus, and polio than parents in western federal states [29].

We found that the proportion vaccinated with a recommended series increased from 74.1% (95%CI: 69.4%–78.2%) for the birth-cohort 1998–2001 to 81.1% (95%CI: 77.4%–84.4%) for birth-cohort 2002–2005 and stayed stable for consecutive birth-cohorts. Our stratified sub-analysis (data not shown) showed the increase between birth-cohorts was larger in western Germany compared to eastern Germany. The increase corresponds well with the introduction of the hexa-valent vaccine in 2001 [9] and thereby show that HBV-vaccination uptake benefit from the use of hexavalent vaccines. Our results indicate, firstly, that a quarter of the birth cohorts prior to 2001 especially in western Germany should not be regarded as vaccinated. This should be taken into consideration for immunization by risk-indication. Secondly, that an additional effort should be done in order to reach the WHO target of 95% vaccinated in these birth-cohorts.

We observed no meaningful difference in adherence in regards to migration background. The point estimated proportion of children and adolescents with a recommended series was lower for children with a two-sided migration background compared with children and adolescents with no- or a one-sided migration background, although the 95%CI broadly overlapped. This result are in line with the results of the Federal Institue of Public Health (Bundeszentrale für gesundheitliche Aufklärung) on the assessment of parents for the need for vaccinations a 2016 survey [29], which revealed that regardless of the migration status parents rate the importance of HBV-vaccination as high. However, in light of our findings indicating a slightly reduced coverage of the complete HBV-vaccination series among children with two-sided migration background—despite similar rates of timely first-dose administration—there is continued evidence that this heterogeneous group requires targeted public health efforts to ensure they fully benefit from the vaccination program in Germany.

As expected, we observed that the proportion of children and adolescents with a timely first dose and recommended series was higher for children who participated in all preventive health examinations until two years of age than those who did not. Correlation analysis showed that it also correlated with age, which can be explained by the increase in the participation of the preventive health examinations over the years [17].

We found only 30.9% of children and adolescents in Germany received the last dose prior to turning 15 months of age. The cumulated delay happened through the delay of the first dose, that only approximately the half of all 3- to 17-year old received and with the following doses. Using health insurance data an increase in a completed vaccination series of 7% points was reported from age 24 months (75.5%) to 36 months (82.2%) for children born in 2016 and later reaching 85% at 72 months [20, 27]. These findings indicate that the finalisation of the series usually happen in the following years of life and by the time the children reach elementary school age. In a low prevalence country, a low proportion of timely administered hepatitis B vaccination may indicate a low risk perception among parents and health care providers. However, a low proportion of timely administered vaccine reduces the effectiveness of the immunisation programme in terms of early-life protection as well as lower reported vaccination coverage rates.

We found a very low proportion (1.33%, 95%CI: 0.80%-2.21%) with a first dose administered too late to be a birth-dose but prior to being able to two months of age. The median number of days until being two months of age was 10 days, range 1–38 days, indicating that the administration of the first dose happened very close to the recommended age. For some participants, the slightly earlier or later administration could be due to individual risk assessment done by their paediatrician, however, we cannot exclude that this finding is due to schedule misalignment or misinterpretation of vaccination guidelines.

### Limitations and strengths

Our study has some limitations that may impact our results. Firstly, we do not have the exact day of birth. We could thus only calculate intervals for being the age of interest for the analysis of timeliness. This leads to less specific estimates that, however, are more sensitive for the children to have gotten a timely dose. This may have led to an overestimation of timeliness in our analysis.

Although we did additional quality checks with the original data collected, 11% of the original sample had no information on vaccinations. This could result in an overestimation of the proportion of children vaccinated, as previous measles outbreak investigation showed that children not presenting a vaccination card were less well vaccinated than children who present with a vaccination card [20, 30]. This overestimation would, however, be similar for the vaccination coverage found using school entry health examinations, using the same exclusion criteria. For the 62 participants with partly unreadable or incomplete vaccination cards 51 had received at least one HBV vaccination and of these 41 had received at least three HBV vaccinations.

Some of our data on socio-demographic and health utilisation characteristics consist of self-reported data that may be biased due to either lack of recall or recall bias leading to misclassification. We do, however, not have any reason to suspect the misclassification in one particular direction. In addition, this would not affect our main results for birth-cohort and place of living. When comparing the included participants with the total study population in the examination we did not find any large differences in the distribution between groups. The small differences observed for migration status can be due to loss of documents such as vaccination cards, which can also be the reason for the difference seen for the utilisation of recommended early childhood development visits, where a higher proportion of included participants (87.1%) had participated in all visits compared to 85.0% of total study population. For both we can expect a lower adherence to vaccination recommendations, which may mean that we have a slight overestimation of the adherence to vaccination recommendation measures.

Despite the limitations our study also has some major strengths enabling us to assess adherence with national recommendations by doctors and parents in a way not done previously. The results are based on a large population-based sample of children and adolescents aged 3–17 years. We could thus analyse not only the number of HBV vaccination doses but also the timeliness of the different doses as well as the type of vaccine given.

## Conclusion and recommendations

We found that most children received at least one dose to initiate the vaccination series within the first 14 months of life. More than 90% of children in Germany aged 3–17 years in 2014–2017 received three or more doses of HBV vaccination. However, the proportion with a recommended series and timely recommended series was substantially lower and parents to children and adolescents living in eastern Germany had a better adherence with STIKO recommendations.

The use of combination vaccines that include hepatitis B alongside other routine childhood immunizations appears to enhance coverage. These findings offer multiple avenues for targeted interventions to improve vaccination uptake.

Efforts are needed to further increase the adherence of vaccination recommendations to reach the WHO target of 95% coverage. In order to close the gap between the proportion of children having received at least one dose of HBV vaccination and the proportion of children with a recommended vaccination series we would recommend doctors to use all opportunities to check both the number of vaccinations and if the two last doses have six months between them. If not, vaccination series should be completed. Moreover, our results suggest that a risk-based vaccination strategy may be needed to fill gaps in coverage especially among birth cohorts prior to 2001 especially in western Germany. We also recommend future research to identify if the 2020 change in STIKO recommendation will help increase adherence and help Germany reach the 95% vaccination coverage. Other initiatives would need to assess how to better reach the unvaccinated young adults.

## Supplementary Information

Below is the link to the electronic supplementary material.

Supplementary Material 1

## Data Availability

The datasets generated and/or analysed during the current study are not publicly available due some access restrictions that apply to the data underlying the findings. The data set cannot be made publicly available because informed consent from study participants did not cover public deposition of data. However, the minimal data set underlying the findings is archived in the ‘Health Monitoring’ Research Data Centre at the Robert Koch Institute (RKI) and can be accessed by all interested researchers. On-site access to the data set is possible at the Secure Data Center of the RKI’s ‘Health Monitoring’ Research Data Centre. Requests should be submitted to the Research Data Centre, Robert Koch Institute, Berlin, Germany (e-mail: fdz@rki.de).

## Footnotes

1 Please note that due to the probability weighing for each strata the sum of the individual strata does not correspond to the overall estimate of the proportion of participants with a timely recommended vaccination series.

## Abbreviations

DPT: Diphtheria, Pertussis and Tetanus
HBsAg: Hepatitis B Surface antigen
HBV: Hepatitis B virus
HCV: Hepatitis C virus
IQR: Interquartile range
KiGGS: German Health Interview and Examination Survey for Children and Adolescents
STIKO: German Standing Committee on Vaccination
WHO: World Health Organization
