# Supplementary material for: Adherence to hepatitis B vaccination recommendations for children and adolescents aged 3 to 17 years in Germany, 2014–2017: results from a cross-sectional national population-based study
Source: BMC Infect Dis. 2026 Jan 16;26:177. doi: 10.1186/s12879-026-12519-z (PMC12849288; doi:10.1186/s12879-026-12519-z)
Supplement: Supplementary file 1 — Supplementary Material 1 [file 12879_2026_12519_MOESM1_ESM.docx]

Supplementary figure 1: Flow-chart participant inclusion and exclusion, N=3567

Number of participants included

n=3165

Number of participants with vaccination book available or unvaccinated without vaccination book

n=3238

Number of participants with at least one missing date for a HBV vaccination

n=73

Number of participants with all vaccination dates unknown

n=14

Number of participants without vaccination book

(n=267) or partly unreadable, incomplete, or lost (n=62)

n=329

Number participants in examination part

N=3567

Supplementary figure 2a: Calculation of birth dose and example thereof

Date of birth (month and year)

Last date of birth month + 2 days

Birth dose: 1st of the birth month until 2^nd^ of the following month and monovalent

Time

First date of birth month

Example birth dose calculation, participant born in March 2001:

2nd April 2001

1st of March 2001

Birth dose: 1st March 2001 to 2^nd^ of April 2001 and monovalent

Supplementary figure 2b: Calculation of timely first dose, catch-up or postpones vaccination series and an example thereof

Age 5 years

Age 15 months

First day of birth month

Last day of birth month

Postponed series: after age 5 years

Catch-up series: after age 14-months

Timely first dose: Age 2-months

Time

*Example: Calculation of timely first dose, catch-up, or postpones vaccination series, DOB March 2001*

Age 15 months

Age 5 years

1st of March 2001

31st of March 2001

Postponed series: after age 5 years: April 2006 or later

Catch-up series: after age 14-months: after 30^th^ June 2002

Timely first dose: Age 2-months: 1^st^ of May – 30^th^ June 2001

Time

Supplementary figure 3: Description of participants with a birth dose given.

*****included as having a timely and recommended series

Number of participants with a polyvalent third dose

1

Number of participants with two monovalent doses only

2

Number of participants with three or four timely and recommended polyvalent doses

**1 ***

Number of participants with polyvalent second dose

4

Number of participants with monovalent second dose given 28-45 days after the birth dose

22

Number of participants with third monovalent dose given <138 days after the second dose

5

Number of participants with third monovalent dose given >=138 days after the second dose

(recommended birth dose series)

15

Number of participants with third monovalent dose given >=138 days after the second dose and prior to 15 months of age

(timely recommended series)

15*

Number of participants with third monovalent dose given 28-45 days after the second dose (0-1-2-12 schedule)

0

Number of participants with birth dose given

43 [1,52%; 1,06-2,18]

Number of participants with second monovalent dose given <28 days after the birth dose

2

Number of participants with four monovalent doses given

2

Number of participants with third monovalent dose given >=138 days after the second dose and prior to 15 months of age

(**timely recommended series**)

5*

Number of participants with third monovalent dose given >=138 days after the second dose

(recommended birth dose series)

5

Number of participants with third monovalent dose given <138 days after the second dose

9

Number of participants with two monovalent doses only

1

Number of participants with second monovalent dose given >45 days after the birth dose

15
